# The Olfactory Bulbectomy Model of Depression: Brief History, Current Status and Critique

**DOI:** 10.3390/brainsci15080775

**Published:** 2025-07-22

**Authors:** David Coppola

**Affiliations:** Department of Biology, Randolph-Macon College, Ashland, VA 23005, USA; dcoppola@rmc.edu

**Keywords:** affective disorders, animal models, validity, limbic system, behavior

## Abstract

In the last several decades, a number of animal models of neurological diseases have been proposed and validated to one degree or another. This review focuses on the olfactory bulbectomized rodent as a model of major depression, a disorder that, because of its prevalence, has been called the “common cold” of neurological diseases, though the disability it causes is far more profound. After describing the method, a brief history of this model and the various validity claims made for it are discussed. Though a legion of physiological and biochemical sequelae of bulbectomy and other animal models of depression have been reported, the focus of this review is behavioral. Therefore, the neurochemical and molecular aspects of the depression models mentioned in this review will not be explored in depth. Lastly, unresolved questions posed by the bulbectomy model are considered along with its utility in the study of other neurological diseases and its future prospects.

## 1. Introduction

Depression, a multifaceted psychiatric illness, is the second leading cause of disability worldwide, striking up to 5% of adults [1]. The symptoms include feeling sad, irritable or “empty” for weeks or more, accompanied by a loss of pleasure in the activities that the sufferer previously enjoyed. There is an established link between depression and suicide, increasing the urgency for effective and fast-acting treatments [1]. Unfortunately, the etiology of depression is not well understood, and the current treatment options, which include various psychological approaches and antidepressant medications, are too often ineffective [2,3].

Animal models, which have been so useful in studying other disorders, have also been at least partially validated (perhaps surprisingly) for affective disorders like depression [4]. It has been proposed that a valid animal model should have all of the following characteristics: similar symptoms to the corresponding human disorder; treatments that normalize symptoms in humans should also normalize symptoms in the model (and the reverse); conditions that bring on depression (e.g., stress) in humans should also bring on depression in the model; and finally, the disorder in humans should have the same or similar physiological and biochemical corollaries as those in the model [5,6].

Two kinds of animal models of depression can be distinguished: The first type emphasizes construct validity and can be used, in theory, to understand the neurological underpinnings of the disorder. The second type of model emphasizes predictive validity and seeks to identify new antidepressant drugs. Some models of this latter type are appropriate for identifying slow-acting antidepressants, like many from the current pharmacopeia of depression drugs. Other models can be used to identify fast-acting antidepressants [7]. These are not mutually exclusive categories. Thus, a single model may work for both types of inquiry.

For obvious reasons—reasonable phylogenetic similarity, economy, etc.—the field has been dominated by rodent models, a large sample of which can be found in Table 1. Virtually all of the models included in the table have been validated by displaying one or more of the core symptoms of depression, including anhedonia, despair, psychomotor abnormalities, etc. Most depression symptoms in these animal models can be normalized with chronic and sometimes acute treatment with classical antidepressant drugs.

This review will focus on the olfactory bulbectomized (OBX) rodent, one of the oldest animal models of depression, because it is still considered the gold standard with regard to predictive validity and because it is experiencing something of a revival [3,8,9,10]. A number of excellent and exhaustive reviews of the older literature establishing OBX as a model of depression are available [11,12]. By contrast, the goal here is to provide a brief history of the model and consider its current status and future prospects. Throughout this review, behavior will be emphasized.

**Table 1:** Animal models of depression.

Type	Description
*Stress Induced*	
Learned helplessness [13]	Unavoidable shock leading to failure to escape avoidable shock.
Restraint [14]	Chronic exposure to escape-proof confining space.
Chronic mild stress [6]	Unpredictable, mildly stressful experiences, e.g., tilted cage, wet bedding.
Social defeat [15]	Placement in home cage of dominant resident followed by chronic sensory exposure to resident.
Early life adversity [16]	Enforced maternal separation for extended periods.
*Biologically Induced*	
Bulbectomy [11]	Surgical ablation of olfactory bulbs bilaterally.
Neuroinflammation [17]	Injection of lipopolysaccharide, triggering immune response.
Corticosterone admin [18]	Injection or imbibition of “stress hormone.”
Neurocircuit manipulation [19]	Optogenetic manipulation of neural circuits, e.g., BNST.
Genetic manipulation [20]	Molecular knockout of neurotransmitter, receptor, or reuptake pump.

## 2. The Birth of a Model

J. B. Watson, the father of American behaviorism, may have been the first person to comment on the behavioral characteristic of the OBX rat. The subjects in his studies of maze running were found to be “irritable and pugnacious” [21]. Since then, a spate of studies, the vast majority in rodents, have documented an OBX behavioral phenotype termed the “bulbectomy syndrome” [11,12]. The behavioral changes that accrue from OBX will be discussed in detail below.

It appears that Van Riezen and colleagues [22] were the first to suggest the OBX rat as a model of depression based on the observation that antidepressant drugs normalize syndromic behavior [23,24]. For example, a rat made hyperlocomotive by OBX can be returned to the normal levels of activity with the application of classical antidepressant drugs [22].

## 3. The Procedure

The procedure has been described many times, justifying what will be a cursory description here [12,25,26]. Briefly, the subject is deeply anesthetized and placed in a stereotax. Hair on the dorsal skull is shaved, after which germicide is applied to the shaved area. An incision is made in the skin, exposing an area of skull at the frontonasal suture. Two craniotomies, 1 mm in diameter, are made with a dental drill, targeting the skull overlying the olfactory bulbs. Then, each bulb is aspirated using a narrow cannula attached to a vacuum pump. Care must be taken to drive the cannula ventrally until it contacts the cribriform plate, lest viable bulbar tissue be left behind. The cavity created by aspirating the bulb is packed with gel foam; the craniotomy holes are filled with bone wax, and the incision is closed with suture or staple. Finally, the wound is dressed with antibiotic ointment, and the subject is placed in a warm chamber until upright, after which it is returned to its home cage, where it receives analgesic medicine.

This procedure has several shortcomings as a reliable method of producing a depression model: First, it is difficult to completely ablate the bulbs because of their morphology [25]. This is problematic because the olfactory system is well known for its ability to regenerate and to successfully process odor information even in a radically reduced state [27,28]. Thus, to the extent that anosmia is a necessary outcome of the procedure, the surgery described above often fails. Moreover, it is laborious and difficult to histologically confirm complete resection of olfactory bulbs. Second, damage to the accessory olfactory nucleus and frontal cortex are often unintended sequelae of the surgery. Third, such a traumatic insult to the brain triggers homeostatic responses that manifest themselves across different time frames, making the model temporally unstable [25]. Fourth, the entire forebrain circulatory system is compromised by the surgery, likely affecting far-flung brain areas in negative ways.

The following recommendations are offered with the goal of making the surgery more targeted, effective and humane: 1. Investigators should work closely with the veterinarian on their IACUC committee to find a drug combination that reliably achieves surgical plane of anesthesia for mice and rats. 2. Sterile technique should be used to avoid infection. 3. Shams that experience all the steps described above, including craniotomy but not perforation of meninges, should be used as controls. 4. Aspiration pressure should be minimized to avoid collateral damage to adjacent brain areas. Specifically, the diameter of the aspiration cannula should be substantially smaller than the diameter of the craniotomy holes. 5. Analgesic treatment should be continued for a minimum of three days after surgery or longer if deemed necessary by the local IACUC. 6. The extent of lesion to the bulb and the presence of any collateral brain damage should be established with postmortem histology, typically using Nissl staining. Adequate sampling should be used to allow detection of residual olfactory glomeruli and small instances of collateral brain damage.

## 4. Behavioral Sequelae of OBX

A number of behavioral anomalies have been reported following the OBX procedure in rodents [12]. However, only naturalistic behaviors that are likely to provide survival value and thus be underpinned by phylogenetically ancient brain circuits will be discussed here [29,30]. Excluded are the so-called “despair” tests, such as the forced swim and tail suspension tests [31,32]. Recently published data on CD-1 mice from the author’s lab will be used to illustrate some of the most frequently studied behavioral sequelae of OBX in rodents [9]. For the most part, these data support previous reports [11,12]. However, when differences and unresolved controversies arise, they will be highlighted.

### 4.1. Locomotion

**Figure 1:**
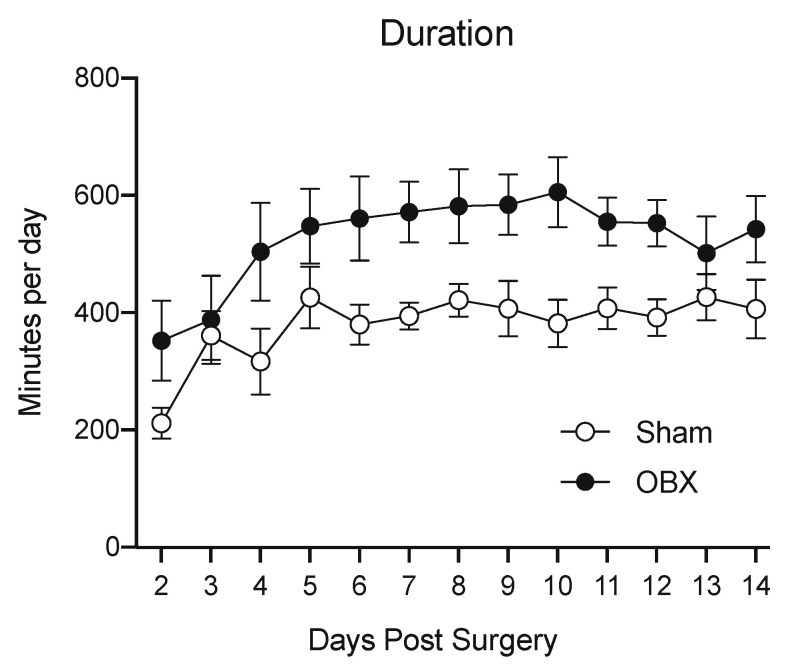
Means (±SEMs) of daily voluntary wheel running durations by mice in the home cage. For sham group, *n* = 7. For OBX group, *n* = 8. During the first two weeks after surgery, OBX subjects ran for longer durations (F(1, 15) = 5.9; *p* < 0.03) than sham controls. Day of the study (i.e., time) was a significant contributing factor in the analysis of duration (F(4.3, 63.5) = 4.9; *p* < 0.002). Duration measures were not normalized for body weight. Modified from [9].

Interestingly, hyperlocomotion is one of the earliest, most salient and most utilized endpoints in the studies of the OBX rodent model of depression, as noted above [32]. Hyperlocomotion, which is defined as motor activity leading to changes in the location of the whole body in external space, contrasts with hyperactivity that includes non-motor components, including inattention, agitation and impulsivity [11,12]. This enhanced activity was initially thought to be limited to stressful environments, like “open field” arenas, but more recently has been found to occur even in the home cage [9,33,34]. For example, in our studies of the CD-1 mouse (Figure 1), OBX subjects ran on the wheels placed in their home cages for significantly longer daily durations than sham mice. This difference appeared three days after the OBX surgery and lasted throughout the two weeks of recording activity.

Though hyperlocomotion in the OBX rodent has been known for more than 50 years, its cause remains a mystery. That it is not a response to anosmia alone is supported by studies showing that peripherally produced anosmia does not cause hyperlocomotion [12,35,36,37]. Song and Leonard [12] have speculated that the OBX rodent’s hyperlocomotory response to a novel environment, like the open field, may be the result of a reduced ability to habituate. However, it is unclear how this idea would pertain to hyperlocomotion measured in familiar environments like the home cage.

### 4.2. Aggression

**Figure 2:**
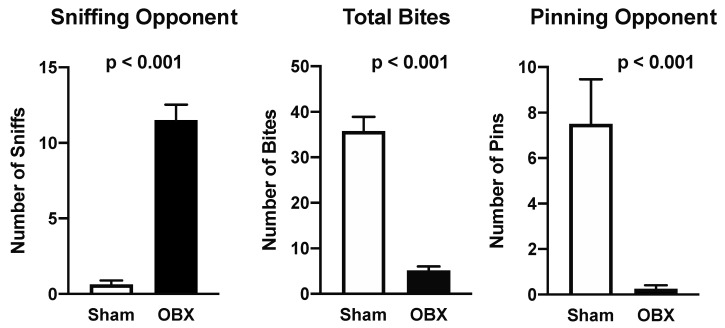
Means (±SEMs) of behavioral events by resident mice in home cages toward intruder mice. For shams, *n* = 8. For OBX mice, *n* = 8. Probabilities are from Mann–Whitney tests and are comparison-wise. Note: The observers performing the scoring were blind to surgery type: sham or OBX (modified from [9]).

Depending on the context and species (rat or mouse), OBX can cause an increase or decrease in aggression. For example, OBX increases mouse-killing behavior and mother–young cannibalism in rats [38,39]. In contrast, we used the “intruder” test, an index of territorial aggression, to show that OBX mice are far less aggressive than control mice (Figure 2) [9]. OBX mice pinned and bit their intruder substantially less than shams. Indeed, there were so few acts of aggression by the OBX mice that they could justifiably be described as “non-aggressive”.

**Figure 3:**
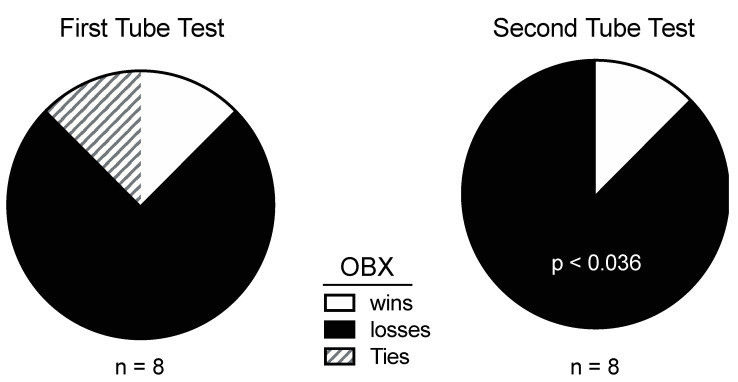
Wins, losses and ties are shown for replicate tube tests pitting shams against OBX mice. Opponents were reshuffled between tests. Significance level in second test is from the binomial test (from [9]).

In order to probe a different dimension of aggression other than territorial defense, we used the “tube test”, in which mice meet snout to snout inside a narrow tube to vie for dominance by pushing their opponent out. This test is widely used to establish dominance hierarchies [40]. In repeated bouts, sham mice were far more likely to push their OBX opponent out of the tube than the reverse (Figure 3). Thus, on two different measures of aggression, OBX mice were shown to be far less aggressive than control mice. These results largely agree with the published literature on intermale aggression in OBX rodents [41,42]. The near elimination of aggression is more parsimoniously attributed to a lack of odor cues that identify gender rather than a depressed state, a conclusion supported by greater sniffing by the OBX subject (Figure 2) [43,44].

### 4.3. Pleasure Taking

**Figure 4:**
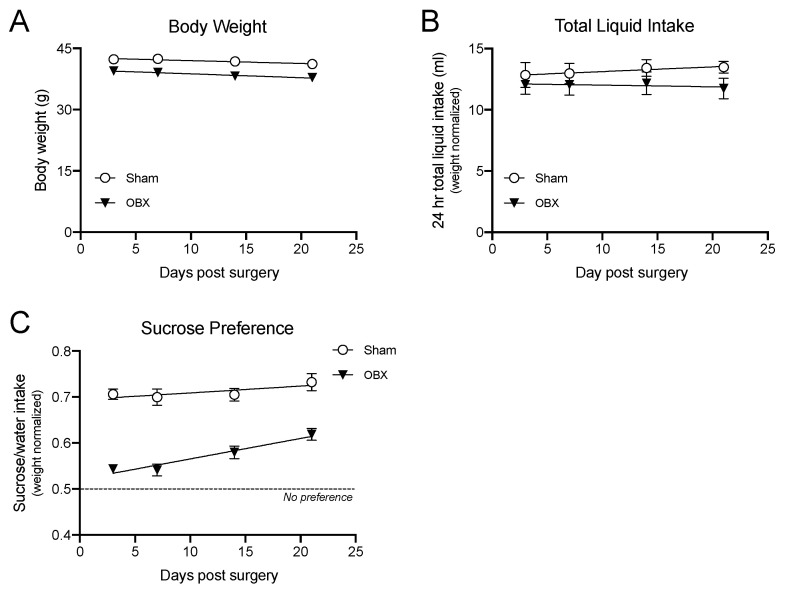
In sham (*n* = 8) and OBX mice (*n* = 8), means (±SEMs) are plotted for (**A**) body weight; (**B**) total liquid consumption; and (**C**) weight-normalized sucrose preference. Tests were conducted on the 4th, 7th, 14th and 21st days after surgery. As body mass and thirst can confound the results of this test, we measured body weight and recorded total liquid intake by subjects on each day of this test. We compared body mass for sham and OBX mice across time using a two-way mixed-model ANOVA. OBX mice had lower body weights than sham controls (F (1, 14) = 8.1; *p* < 0.01), and weight declined across time points for all mice (F (3, 42) = 7.6; *p* 0.0003). Surgery type and time did not interact (F (3, 42) = 0.4; *p* > 0.7). We also compared total liquid intake differences between groups and across time and found no effect of surgery type (F (1, 14) = 2.7; *p* > 0.12) or time (F (3, 42) = 0.09; *p* > 0.9) on total liquid intake. Given the body weight difference between surgery types and across time, we analyzed the dependent variable: sucrose intake/water intake normalized for body weight. Both sham and OBX mice displayed a preference for sucrose over water at all time points. Again, using a two-way mixed-model ANOVA, we observed a main effect for surgery type (F (1, 14) = 168.6; *p* < 0.0001) and time (F (3, 42) = 7.17; *p* < 0.0005) but no significant interaction (F(3, 42) = 1.70; *p* > 0.18). Holm–Sidak’s multiple comparison tests showed that sucrose preference was significantly greater for sham than OBX subjects at each time point (all comparisons *p* < 0.001 or less). Finally, we compared sucrose preference medians to 0.5 (the theoretical value under the null hypothesis) for each group using one-sample Wilcoxon signed-rank tests. All values confirm significant sucrose preference with probabilities ranging from *p* < 0.02 to 0.008 (used with permission [9]).

Anhedonia, the loss of pleasure-taking, is a hallmark symptom of depression in humans. Sucrose preference is a behavioral test used in animals that has face validity as a measure of anhedonia [45,46]. We employed this test in the home cage of OBX and sham mice on four occasions during the first three weeks after OBX or sham surgery (Figure 4). At every timepoint, including the earliest, three days post-surgery, OBX mice showed significantly less preference for sucrose than shams (Figure 4). However, OBX mice preferred sucrose to water at all time points, a preference that increased significantly over time (Figure 4). Thus, if sucrose preference is a legitimate pleasure-taking behavior, then OBX mice are not completely anhedonic, and their level of anhedonia decreases over time after surgery. These results largely agree with the published literature on sucrose preference in the OBX rodent [3,47,48].

### 4.4. Learning and Memory

Cognitive functioning in OBX rodents has been extensively studied. For example, compared to controls, the OBX rat displays decreased spatial navigation ability in the Morris water maze [49,50]. They have also shown memory and learning deficits using conditioned taste aversion [51] and both passive [52] and active [53] avoidance tests.

**Figure 5:**
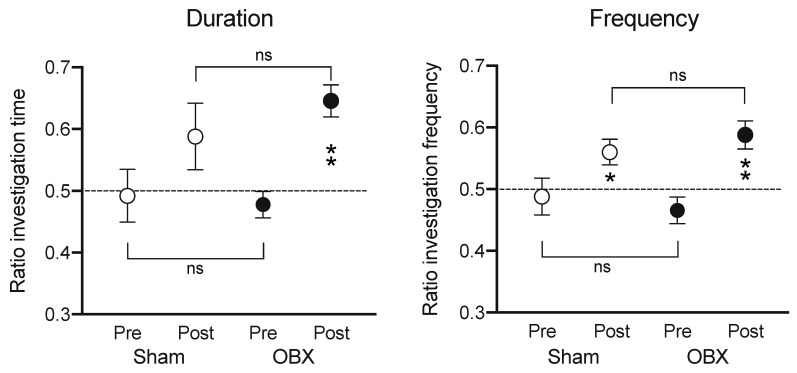
Means (±SEMs) are illustrated for duration (**left**) and frequency (**right**) of novel object investigation, expressed as a ratio of total investigation time. Pre-test and post-test (novel object introduced after 24 h) values are shown for sham (*n* = 5) and OBX mice (*n* = 9). Asterisks denote significance from no preference (null hypothesis = 0.5) using Wilcoxon test (* *p* < 0.05; ** *p* < 0.01) Brackets show results of Mann–Whitney test. Sham and OBX mice displayed significantly similar increased attention to the novel object. Subjects demonstrated no preferences in the pre-test, indicating a lack of position bias (Wilcoxon W = −1; *p* > 0.9—sham; Wilcoxon W = −18; *p* > 0.3—OBX). Analysis of behavior during the post-test revealed that OBX mice exhibit a preference for the novel object in both investigation duration (Wilcoxon W = 36; *p* < 0.008) and frequency (Wilcoxon W = 28; *p* < 0.016). Sham mice approached the one-tailed alpha levels (duration: Wilcoxon W = 11, *p* < 0.09; frequency: Wilcoxon W = 10, *p* < 0.06), indicating a possible preference for the novel object. Behavioral measures for sham and OBX mice were similar during the post-test (Mann–Whitney duration: U = 17.5, *p* > 0.5; frequency: U = 16.5, *p* > 0.4.). ns = nonsignificant. From [9].

Our laboratory tested the effects of OBX on learning and memory in the mouse, using the widely employed “novel object test” [54,55]. This is not a test of spatial ability and does not require any punishment for errors (e.g., electric shock), like certain other tests (Figure 5). Both sham and OBX mice displayed increased duration and frequency of investigation toward a novel object, and their data were statistically indistinguishable. Thus, for at least one test of learning and memory, OBX mice fail to show any deficit. This null result contrasts with another report that found deficits for the novel object test in OBX mice, although using different parameters [56].

### 4.5. Fearfulness

**Figure 6:**
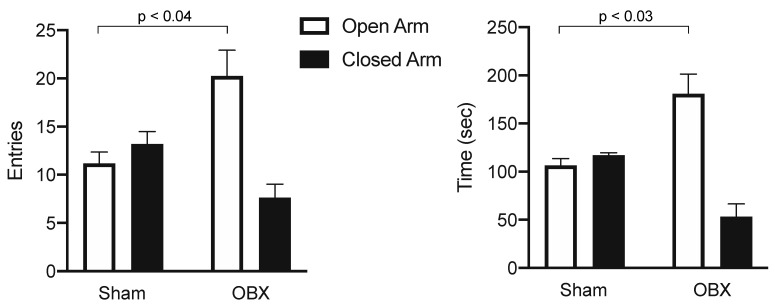
Means (±SEMs) are illustrated for number of entries (**left**) and investigation time (**right**) in the open and closed arms of an elevated plus maze for sham (*n* = 5) and OBX (*n* = 8) mice. OBX mice displayed a preference for the open arms, while shams displayed a non-significant preference for the closed arms. Probabilities are derived from Mann–Whitney tests. Sham mice made more visits (Wilcoxon Signed Rank: W = 15; *p* < 0.03) to the closed arms and spent more time there (Wilcoxon Signed Rank: W = 15; *p* < 0.03 one-tailed). In contrast, OBX subjects made more visits (Wilcoxon Signed Ranks: W = 36; *p* < 0.004 one-tailed) and spent more time (Wilcoxon Signed Rank: W = 36; *p* < 0.004 one-tailed) in the open arms. Consistent with these observations, the number of entries (Mann–Whitney: U = 5.5; *p* < 0.04 two-tailed) and time spent (Mann–Whitney: U = 5; *p* < 0.03 two-tailed) in the open arms of the maze were greater for OBX than sham subjects (from [9]).

In humans, anxiety is the most common comorbidity of depression [57]. Classically, anxiety or fearfulness has been assessed in rodents using the elevated plus maze, among other methods [58]. The more entries and time spent in the walled-off arms of the maze compared to the open arms, the more anxious the subject is thought to be. When we compared the number of entries and time spent in the open and walled-off portions of the plus maze, we found that, unlike shams, OBX mice made significantly more entries and spent significantly more time in the open than in the walled-off arms of the maze (Figure 6). This finding in the CD-1 mouse is consistent with the observations made in the rat, suggesting that the OBX surgery has an anxiolytic effect on rodents [59,60].

## 5. Physiology

Though behavior is the focus here, it should be emphasized that changes in various hormones, neurotransmitters and components of the immune system accompany OBX in the rodent [11,12]. Importantly, many of these changes mimic those seen in the depressed human patients and may serve as biomarkers of the disease [12]. Moreover, many biological sequelae of OBX are normalized by antidepressant drugs, in most cases only through chronic dosing. This latter characteristic mimics the behavior of nearly all classical antidepressants used clinically. Whatever the merits of the OBX model of depression in the behavior realm, it has made contributions to our understanding of the mechanistic pathways and chronic treatment responses of depression. The list of physiological and molecular sequelae of OBX in rodents discovered so far has been extensively reviewed [11,12].

## 6. Why Should OBX Cause a Depression-like State?

**Figure 7:**
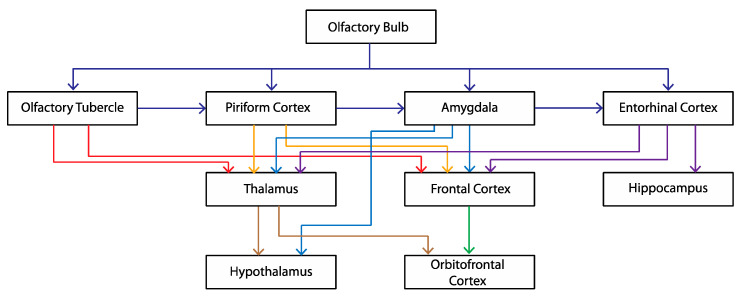
Olfactory bulb projection (redrawn from [3]).

An anatomical answer to this question lies in the close association between the olfactory system and brain areas involved in emotions and mood (Figure 7). Limbic system components, including the orbitofrontal cortex, cingulate cortex, hippocampus and amygdala, are connected in a functional network to the olfactory system such that a severe challenge to the integrity of olfaction will have a significant effect on the operation of these and other limbic structures. The close connection between olfaction and depression is also supported by clinical data: The depressed population, compared to the non-depressed population, has diminished ability in the tests of odor threshold [61,62,63,64], discrimination [65,66] and odor identity [66,67]. Reciprocally, patients with olfactory dysfunction are far more likely to be depressed than the general population [68]. Given olfaction’s role in “quality of life” attainment, including protection from noxious chemicals, enjoyment of food and drink, and memory processes, it is reasonable to consider a causal link between olfactory dysfunction and clinical depression [68].

## 7. Validity of the OBX Model of Depression

### 7.1. Face Validity

Here we ask, does the model have similar symptoms to the corresponding human disorder? As shown in Table 2, which compares the symptoms of depression to the sequelae of rodent OBX, the results are mixed [57]. Once you eliminate the symptoms for which there are no analogies in rodents (e.g., depressed mood, suicidality), you are left with two similarities: “change in activity” (with the direction unspecified in humans) and anhedonia. The activity piece is unconvincing, given that marked hyperlocomotion is the invariant result of OBX in rodents, while the depressed patient often presents with profound hypoactivity [57]. The co-expression of anhedonia in human and non-human animals is more compelling since it characterizes all forms of clinical depression [57]. However, this comparison requires the assumption that the level of sucrose preference in an animal model is a legitimate equivalent to pleasure-taking in humans.

### 7.2. Construct Validity

Here we ask if the model has similar neurobiological bases to the human disorder. An in-depth analysis of construct validity is beyond the scope of this behaviorally focused review. Briefly, a problem exists in assigning construct validity to any model, given that the neurobiological bases of depression remain elusive. Depression is thought to result from a web of causal factors with a unifying theme of maladaptive responses to chronic or repeated stressors, especially by the immune and endocrine systems [69]. In this context, there have been a number of parallels reported between the OBX rodent and the depressed human patient. For the immune system, these include a decline in the neutrophil phagocytic reaction [70] and a decline in mitogen-stimulated lymphocyte proliferation [71], to list but two [11]. For the endocrine system, hypersecretion of cortisol was an early report in the depressed patient and OBX rodent, suggesting a chronic state of stress in both [72,73].

Many antidepressant drugs target specific monoamine neurotransmitter systems consistent with the longstanding “monoamine hypothesis” of depression. Thus, it is interesting, with respect to the noradrenergic system, to note that both depressed patients and OBX rats show a similar compensatory increase in the function and number of adrenoceptors [74,75]. In a similar vein, the brain content of serotonin and one of its metabolites are reduced in both depressed patients and OBX rodents [12,76]. Numerous other parallels between depressed patients and OBX rodents have been reported for other neurotransmitter systems, a topic that has been thoroughly reviewed [11,12].

Unfortunately, the many neurobiological parallels between the OBX rodent and the depressed patient fail to establish a construct validity, given that it is unclear if these are “causal factors, correlates, or consequences of depression” [77].

### 7.3. Predictive Validity

This form of validity refers to a model’s ability to predict future outcomes. In the case of the OBX model, it usually refers to its accuracy in screening for new antidepressant drugs. The predictive validity of the OBX model of depression is generally considered peerless [8]. Indeed, more than 60 known or potential antidepressants have been tested for their ability to normalize OBX syndromic sequelae at the level of behavior, physiology and biochemistry [11,12]. As noted previously, hyperlocomotion has been the most commonly measured behavioral sequela of the model in drug screens. Different classes of antidepressants have been shown to normalize hyperlocomotion (reduced to the level of controls) if dosed chronically, but not acutely. This quality of the model—the need for chronic treatment—adds to its validity, given the well-known delay in the effectiveness of most antidepressants used clinically. Nevertheless, there have been false positives and false negatives in the tests of potential antidepressants, and the model’s ability to screen for fast-acting antidepressants, a major goal of the current drug-discovery efforts, has been questioned [7].

## 8. OBX Model of Depression: Remaining Questions

Summarizing the validity claims for the OBX model of depression does not support a great deal of confidence in the method. The model has moderate face validity—assuming sucrose preference is a valid index of anhedonia. However, rodents can detect the smell of sugar, raising the possibility that anosmia caused by OBX reduces the avidity for sucrose solutions [9].

The model has questionable construct validity and no etiological validity. The one area where it stands out is its predictive validity in drug screens. However, many of the drugs tested against the model have the same or similar biochemical action (e.g., selective serotonin reuptake inhibitors). Given this similarity of action, they do not provide independent validation of the model [78]. In addition, screens for new and fast-acting antidepressants using the model have produced false positive and negative results [7].

Another unresolved question concerning the model is that the mechanism underlying the most commonly measured sequela of OBX—hyperlocomotion—and why it is normalized by most antidepressants remains a mystery. While this is not an empirical problem—any consistent change in behavior that identifies effective antidepressant drugs could be used in a screen—this deficit in theoretical understanding remains a cause of concern. Hyperlocomotion brought on by OBX is not preserved across taxa. It is a marked and consistent response to OBX in rats and mice but not in non-human primates, nor is depression typically associated with hyperlocomotion in humans [57]. Furthermore, a recent report observed a diminished locomotory response to olfactory bulb ablation in zebrafish [10]. These facts speak to our poor understanding of the most basic features of the model.

Yet another concern is that OBX sequelae that are due to anosmia have never been satisfactorily distinguished from those due to other causes (e.g., damage to the limbic system). The literature suggests this dichotomy between olfactory versus limbic system causes has been resolved, but doubts remain [9]. This uncertainty emerges from the fact that the method used in previous studies to produce peripheral anosmia—zinc sulphate lavage of the nasal cavity—which has failed to produce the same sequelae as OBX, is problematic [79]. Zinc sulphate is a toxic substance that kills some olfactory receptors when infused into the external nares. Unfortunately, some of this chemical is invariably swallowed by the subjects during the lavage procedure, leading to a malaise lasting for days (and causing death in some subjects). Thus, one does not know in the subsequent behavioral tests on the surviving subjects if the results are influenced by olfactory deficits or malaise or both. Moreover, when confirmed with postmortem histology, the lavage procedure rarely, if ever, achieves complete coverage of the olfactory receptors, leaving some viable olfactory receptors intact [80]. When rigorously tested using gold-standard olfactometry, the procedure has been shown to produce only temporary sensory loss lasting, at most, a few days due to the olfactory mucosa’s ability to regenerate [80,81]. These considerations are compounded by the fact that “anosmia” rarely has been confirmed in previous studies. Together, these points raise doubts as to whether the role of olfactory loss in the emergence of OBX syndrome has been definitively tested.

Finally, the brain-wide, temporally unstable and non-specific effects of OBX have long been known [25]. The procedure compromises the blood–brain barrier and causes profound expansion of the lateral ventricles, thinning of the cortex and hippocampal neuron loss, effects that are not normalized by antidepressants [82]. Furthermore, on the issue of non-specificity, a recent study has shown that when gamma waves produced by the olfactory bulbs are electronically interfered with, anhedonia, but not hyperlocomotion, is the result [3]. This suggests that the different sequelae of OBX in rodents may have different causes.

## 9. Conclusions

OBX is one of the oldest animal models of depression and is still considered, by some investigators, to be the gold standard of predictive validity (Table 3). Nevertheless, its promise as a method of high-throughput drug screening never seems to have been realized, and it is unclear if the model has been responsible for the discovery of even a single novel antidepressant currently in clinical use. The value of the OBX model may be becoming academic, given the current lack of a robust depression drug-discovery pipeline, which has been blamed, in part, on the lack of valid animal models [78]. In particular, the use of rodents has been criticized as an inappropriate taxon. This may be the case.

At the behavioral level, the OBX mice in our tests did not subjectively appear to be depressed. Rather, they appeared tireless, preoccupied with exploration, less anxious than sham controls and non-aggressive. In other words, not reminiscent of the symptoms of depression listed in the DSM-V, even those that can be mapped onto rodent behavior (Table 2).

In addition to major depression, the OBX model has been used in research on anxiety disorder [8], attention deficit hyperactivity disorder [83] and Alzheimer’s [82]. All of these uses suffer from the same shortcomings of the model that have been emphasized in this review: They assume, incorrectly, that bulbectomy surgery is usually complete and, in any case, simple to verify; brain areas adjacent to the bulbs are rarely damaged by OBX; anosmia can be excluded as a cause of the behavioral sequelae of the surgery; after some short recovery period, the behavioral and physiological sequelae of the surgery remain stable; and, perhaps most importantly, OBX is the equivalent of removing a component from a device, the results being predictable and invariant. Taken together, these considerations lead to the conclusion that the OBX rodent is as complex, temporally unstable and misunderstood as the disorders it has been purported to model, making it a questionable choice for the study of neurological disease.

## Figures and Tables

**Table 2:** Major depression.

Symptoms	Analogue in OBX Rodent
Depressed mood	No analog
Diminished interest/pleasure in activities	Decrease in sucrose preference
Significant increase or decrease in weight	Little or none
Change in sleep	Little or no change in circadian rhythm
Change in activity level (locomotion)	Increased in open field and home cage
Decreased energy	None
Feelings of worthlessness or guilt	No analog
Decreased ability to concentrate	Unknown
Suicidality	No analog

**Table 3:** OBX Model Summary.

Strengths	Limitations	Open Questions
Unmatched predictive validity useful in new drug screens.	Surgery causes brain-wide effects, compromising specificity.	Basis of hyperlocomotory response to surgery is unknown.
Model’s chronic state of stress, thought to be the basis of MDD.	Construct and face validity questionable.	Role of anosmia in behavioral sequelae remains unresolved.
Model’s need for chronic dosing of classical antidepressants.	Permanent effects do not model relapsing/remitting nature of MDD.	Effects on limbic system need clarification.

MDD = Major Depressive Disorder.

## Data Availability

Not applicable.
